# Poly[aqua­[μ_2_-1,1′-(butane-1,4-di­yl)diimidazole](μ_2_-naphthalene-1,4-dicarboxyl­ato)nickel(II)]

**DOI:** 10.1107/S1600536808024008

**Published:** 2008-08-06

**Authors:** Xian-Zhi Zou

**Affiliations:** a Department of Chemistry, College of Chemistry and Biology, Beihua University, Jilin City 132013, People’s Republic of China

## Abstract

In the title compound, [Ni(C_12_H_6_O_4_)(C_10_H_14_N_4_)(H_2_O)]_*n*_, the coordination polyhedron around each Ni^II^ atom is a distorted *cis*-NiN_2_O_4_ octa­hedron. The naphthalene-1,4-dicarboxyl­ate and 1,1′-(butane-1,4-di­yl)diimidazole ligands bridge the Ni centres to form a two-dimensional (4,4)-network, and O—H⋯O hydrogen bonds complete the structure.

## Related literature

For general background, see: Batten & Robson (1998 ▶). For a related structure, see: Ma *et al.*, (2003 ▶).
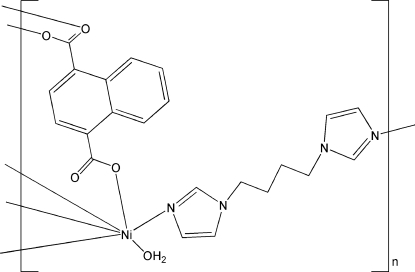


## Experimental

### 

#### Crystal data

[Ni(C_12_H_6_O_4_)(C_10_H_14_N_4_)(H_2_O)]
*M* *_r_* = 481.15
Monoclinic, 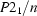

*a* = 12.4213 (12) Å
*b* = 13.2543 (13) Å
*c* = 13.4328 (13) Å
β = 107.361 (2)°
*V* = 2110.8 (4) Å^3^
*Z* = 4
Mo *K*α radiation
μ = 0.96 mm^−1^
*T* = 293 (2) K
0.19 × 0.17 × 0.15 mm

#### Data collection

Bruker APEX CCD diffractometer
Absorption correction: multi-scan (*SADABS*; Bruker, 1998 ▶) *T*_min_ = 0.827, *T*_max_ = 0.866
11720 measured reflections
4157 independent reflections
2982 reflections with *I* > 2σ(*I*)
*R*_int_ = 0.063

#### Refinement

*R*[*F* ^2^ > 2σ(*F* ^2^)] = 0.049
*wR*(*F* ^2^) = 0.097
*S* = 1.05
4157 reflections
297 parameters
3 restraints
H atoms treated by a mixture of independent and constrained refinement
Δρ_max_ = 0.54 e Å^−3^
Δρ_min_ = −0.40 e Å^−3^

### 

Data collection: *SMART* (Bruker, 1998 ▶); cell refinement: *SAINT* (Bruker, 1998 ▶); data reduction: *SAINT*; program(s) used to solve structure: *SHELXS97* (Sheldrick, 2008 ▶); program(s) used to refine structure: *SHELXL97* (Sheldrick, 2008 ▶); molecular graphics: *SHELXTL* (Sheldrick, 2008 ▶); software used to prepare material for publication: *SHELXTL*.

## Supplementary Material

Crystal structure: contains datablocks global, I. DOI: 10.1107/S1600536808024008/hb2770sup1.cif

Structure factors: contains datablocks I. DOI: 10.1107/S1600536808024008/hb2770Isup2.hkl

Additional supplementary materials:  crystallographic information; 3D view; checkCIF report

## Figures and Tables

**Table 1:** Selected bond lengths (Å)

Ni1—O1*W*	2.125 (2)
Ni1—O2	2.040 (2)
Ni1—O1^i^	2.116 (2)
Ni1—O3^i^	2.347 (2)
Ni1—N1	2.060 (3)
Ni1—N4^ii^	2.099 (3)

Symmetry codes: (i) 
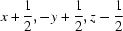
; (ii) 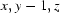.

**Table 2:** Hydrogen-bond geometry (Å, °)

*D*—H⋯*A*	*D*—H	H⋯*A*	*D*⋯*A*	*D*—H⋯*A*
O1*W*—H*W*12⋯O1^iii^	0.819 (16)	1.847 (18)	2.661 (3)	172 (3)
O1*W*—H*W*11⋯O4	0.83 (4)	1.83 (4)	2.651 (3)	169 (3)

Symmetry code: (iii) 
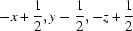
.

**Fig. 1.**
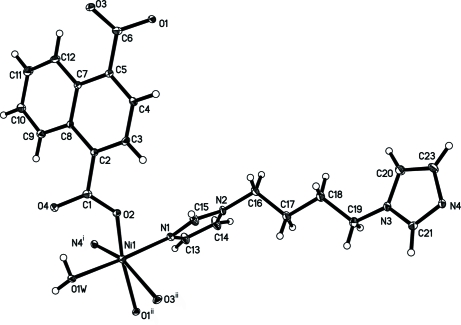
The structure of (I), with displacement ellipsoids for the non-hydrogen atoms drawn at the 30% probability level. Symmetry codes: (i) x, y - 1, z; (ii) 1/2 + x, 0.5 - y, z - 1/2.

**Fig. 2.**
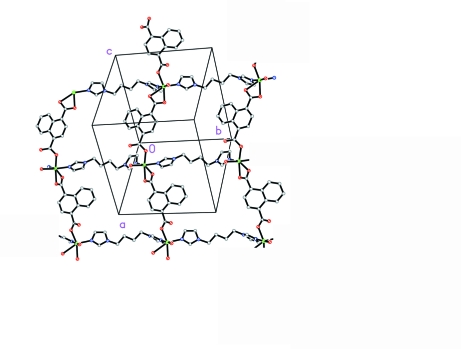
View of part of the polymeric layer structure of (I).

## supplementary crystallographic information

### Comment

Metal-organic frameworks are currently of great interest because of their interesting structures and potential applications. So far, some interesting interpenetrated or entangled metal-organic networks with bis(imidazole)-containing ligands have been documented (Batten & Robson, 1998).  Flexible ligands such as 1,1'-(1,4-butanediyl)bis(imidazole) (*L*) have been less explored to date (Ma *et al.*, 2003). In this work, we selected 1,4-naphthalenedicarboxylic acid (H_2_ndc) and *L* as linkers, generating a new coordination polymer, [Ni(ndc)(*L*)(H_2_O)], (I), which is reported here.

In compound (I) each Ni^II^ atom is six-coordinated by two N atoms from two different *L* ligands, and four O atoms from three carboxylate oxygen atoms (one bidentate, one monodentate) and one water molecule in a distorted cis-NiN_2_O_4_ octohedral coordination sphere (Fig. 1). The two neighbouring Ni^II^ atoms are bridged by the ndc and *L* ligands to form a two-dimensional (4,4) network (Fig. 2) and O—H···O hydrogen bonds arising from the water molecule (Table 2) complete the structure.

### Experimental

A mixture of H_2_ndc (0.5 mmol), *L* (0.5 mmol), NaOH (1 mmol) and NiCl_2_^.^6H_2_O (0.5 mmol) was suspended in 12 ml of deionized water and sealed in a 20-ml Teflon-lined autoclave. Upon heating at 433 K for one week, the autoclave was slowly cooled to room temperature. Green blocks of (I) were collected, washed with deionized water and dried.

### Refinement

The H atoms on C atoms were generated geometrically and refined as riding with C—H = 0.93Å and *U*_iso_(H) = 1.2*U*_eq_(C). The water H atoms were located in a difference Fourier map and refined with the O—H distance restrained to 0.85±0.01 Å.

### Crystal data

**Table:**

[Ni(C_12_H_6_O_4_)(C_10_H_14_N_4_)(H_2_O)]	*F*_000_ = 1000
*M**_r_* = 481.15	*D*_x_ = 1.514 Mg m^−^^3^
Monoclinic, *P*2_1_/*n*	Mo *K*α radiation λ = 0.71073 Å
Hall symbol: -P 2yn	Cell parameters from 4157 reflections
*a* = 12.4213 (12) Å	θ = 1.9–26.1º
*b* = 13.2543 (13) Å	µ = 0.96 mm^−^^1^
*c* = 13.4328 (13) Å	*T* = 293 (2) K
β = 107.361 (2)º	Block, green
*V* = 2110.8 (4) Å^3^	0.19 × 0.17 × 0.15 mm
*Z* = 4	

### Data collection

**Table:**

Bruker APEX CCD diffractometer	4157 independent reflections
Radiation source: fine-focus sealed tube	2982 reflections with *I* > 2σ(*I*)
Monochromator: graphite	*R*_int_ = 0.063
*T* = 293(2) K	θ_max_ = 26.1º
ω scans	θ_min_ = 2.0º
Absorption correction: multi-scan(SADABS; Bruker, 1998)	*h* = −9→15
*T*_min_ = 0.827, *T*_max_ = 0.866	*k* = −16→14
11720 measured reflections	*l* = −16→16

### Refinement

**Table:**

Refinement on *F*^2^	Secondary atom site location: difference Fourier map
Least-squares matrix: full	Hydrogen site location: inferred from neighbouring sites
*R*[*F*^2^ > 2σ(*F*^2^)] = 0.049	H atoms treated by a mixture of independent and constrained refinement
*wR*(*F*^2^) = 0.097	*w* = 1/[σ^2^(*F*_o_^2^) + (0.0253*P*)^2^] where *P* = (*F*_o_^2^ + 2*F*_c_^2^)/3
*S* = 1.05	(Δ/σ)_max_ < 0.001
4157 reflections	Δρ_max_ = 0.54 e Å^−^^3^
297 parameters	Δρ_min_ = −0.40 e Å^−^^3^
3 restraints	Extinction correction: none
Primary atom site location: structure-invariant direct methods	

### Special details

**Table:**

Geometry. All e.s.d.'s (except the e.s.d. in the dihedral angle between two l.s. planes) are estimated using the full covariance matrix. The cell e.s.d.'s are taken into account individually in the estimation of e.s.d.'s in distances, angles and torsion angles; correlations between e.s.d.'s in cell parameters are only used when they are defined by crystal symmetry. An approximate (isotropic) treatment of cell e.s.d.'s is used for estimating e.s.d.'s involving l.s. planes.
Refinement. Refinement of *F*^2^ against ALL reflections. The weighted *R*-factor *wR* and goodness of fit *S* are based on *F*^2^, conventional *R*-factors *R* are based on *F*, with *F* set to zero for negative *F*^2^. The threshold expression of *F*^2^ > σ(*F*^2^) is used only for calculating *R*-factors(gt) *etc*. and is not relevant to the choice of reflections for refinement. *R*-factors based on *F*^2^ are statistically about twice as large as those based on *F*, and *R*- factors based on ALL data will be even larger.

### Fractional atomic coordinates and isotropic or equivalent isotropic displacement parameters (Å^2^)

**Table:**

	*x*	*y*	*z*	*U*_iso_*/*U*_eq_	
C1	0.2969 (3)	0.1196 (2)	0.2222 (3)	0.0208 (8)	
C2	0.2145 (3)	0.1584 (2)	0.2758 (2)	0.0190 (8)	
C3	0.2114 (3)	0.2586 (2)	0.2946 (3)	0.0264 (9)	
H3	0.2583	0.3019	0.2721	0.032*	
C4	0.1392 (3)	0.2986 (2)	0.3472 (3)	0.0238 (8)	
H4	0.1399	0.3677	0.3597	0.029*	
C5	0.0674 (3)	0.2376 (2)	0.3805 (2)	0.0203 (8)	
C6	−0.0034 (3)	0.2829 (2)	0.4420 (3)	0.0218 (8)	
C7	0.0636 (3)	0.1328 (2)	0.3576 (2)	0.0180 (7)	
C8	0.1382 (3)	0.0919 (2)	0.3055 (2)	0.0172 (7)	
C9	0.1306 (3)	−0.0118 (2)	0.2806 (3)	0.0256 (8)	
H9	0.1795	−0.0393	0.2473	0.031*	
C10	0.0536 (3)	−0.0723 (3)	0.3042 (3)	0.0281 (9)	
H10	0.0497	−0.1404	0.2868	0.034*	
C11	−0.0196 (3)	−0.0320 (3)	0.3546 (3)	0.0312 (9)	
H11	−0.0722	−0.0738	0.3706	0.037*	
C12	−0.0157 (3)	0.0664 (2)	0.3806 (3)	0.0257 (8)	
H12	−0.0657	0.0914	0.4141	0.031*	
C13	0.1710 (3)	0.2460 (3)	−0.1581 (3)	0.0335 (10)	
H13	0.1644	0.1937	−0.2056	0.040*	
C14	0.1151 (4)	0.3340 (3)	−0.1787 (3)	0.0430 (11)	
H14	0.0644	0.3537	−0.2418	0.052*	
C15	0.2219 (3)	0.3312 (3)	−0.0198 (3)	0.0310 (9)	
H15	0.2581	0.3510	0.0484	0.037*	
C16	0.1118 (3)	0.4914 (2)	−0.0714 (3)	0.0274 (9)	
H16A	0.1268	0.5024	0.0029	0.033*	
H16B	0.0311	0.4976	−0.1039	0.033*	
C17	0.1715 (3)	0.5711 (2)	−0.1149 (3)	0.0296 (9)	
H17A	0.1498	0.5648	−0.1903	0.036*	
H17B	0.2523	0.5605	−0.0882	0.036*	
C18	0.1434 (3)	0.6771 (2)	−0.0863 (3)	0.0293 (9)	
H18A	0.0622	0.6862	−0.1083	0.035*	
H18B	0.1705	0.6855	−0.0112	0.035*	
C19	0.1971 (3)	0.7560 (2)	−0.1379 (3)	0.0293 (9)	
H19A	0.2754	0.7381	−0.1277	0.035*	
H19B	0.1590	0.7559	−0.2123	0.035*	
C20	0.1050 (3)	0.9021 (3)	−0.0704 (3)	0.0395 (11)	
H20	0.0353	0.8734	−0.0749	0.047*	
C21	0.2746 (3)	0.9270 (2)	−0.0777 (3)	0.0238 (8)	
H21	0.3436	0.9163	−0.0898	0.029*	
N4	0.2477 (2)	1.01080 (19)	−0.0399 (2)	0.0212 (7)	
C23	0.1402 (3)	0.9948 (3)	−0.0365 (3)	0.0378 (10)	
H23	0.0973	1.0419	−0.0137	0.045*	
N1	0.2384 (2)	0.2446 (2)	−0.0581 (2)	0.0224 (7)	
N2	0.1476 (2)	0.38862 (19)	−0.0887 (2)	0.0232 (7)	
N3	0.1920 (2)	0.8579 (2)	−0.0970 (2)	0.0240 (7)	
O1	−0.05860 (18)	0.36339 (16)	0.40707 (17)	0.0227 (5)	
O2	0.3062 (2)	0.17401 (16)	0.14730 (18)	0.0266 (6)	
O1W	0.4733 (2)	0.04150 (17)	0.12115 (19)	0.0208 (6)	
O3	−0.00731 (19)	0.24467 (16)	0.52507 (17)	0.0265 (6)	
O4	0.35191 (19)	0.04171 (16)	0.25344 (18)	0.0257 (6)	
Ni1	0.35134 (4)	0.13565 (3)	0.01819 (3)	0.02231 (14)	
HW12	0.495 (2)	−0.0135 (16)	0.107 (2)	0.020 (10)*	
HW11	0.440 (3)	0.034 (2)	0.166 (2)	0.053 (15)*	

### Atomic displacement parameters (Å^2^)

**Table:**

	*U*^11^	*U*^22^	*U*^33^	*U*^12^	*U*^13^	*U*^23^
C1	0.023 (2)	0.0180 (19)	0.0236 (19)	−0.0040 (15)	0.0094 (15)	−0.0067 (15)
C2	0.025 (2)	0.0174 (19)	0.0172 (18)	0.0032 (14)	0.0099 (15)	0.0011 (14)
C3	0.034 (2)	0.0166 (19)	0.037 (2)	−0.0033 (16)	0.0226 (18)	0.0027 (16)
C4	0.033 (2)	0.0108 (18)	0.033 (2)	0.0005 (15)	0.0184 (18)	−0.0043 (15)
C5	0.024 (2)	0.0201 (19)	0.0198 (19)	0.0018 (15)	0.0110 (15)	0.0017 (15)
C6	0.023 (2)	0.0178 (19)	0.028 (2)	−0.0063 (15)	0.0133 (17)	−0.0083 (16)
C7	0.0207 (18)	0.0162 (18)	0.0187 (17)	0.0009 (14)	0.0085 (14)	0.0007 (14)
C8	0.0238 (19)	0.0136 (17)	0.0162 (17)	0.0012 (14)	0.0088 (15)	0.0009 (13)
C9	0.030 (2)	0.021 (2)	0.030 (2)	0.0018 (16)	0.0157 (17)	−0.0013 (16)
C10	0.034 (2)	0.0147 (19)	0.039 (2)	−0.0018 (16)	0.0167 (19)	−0.0041 (16)
C11	0.037 (2)	0.022 (2)	0.041 (2)	−0.0094 (17)	0.021 (2)	−0.0018 (17)
C12	0.030 (2)	0.025 (2)	0.028 (2)	−0.0024 (16)	0.0182 (17)	−0.0027 (16)
C13	0.047 (3)	0.023 (2)	0.029 (2)	0.0090 (18)	0.0076 (19)	−0.0050 (17)
C14	0.057 (3)	0.035 (2)	0.026 (2)	0.019 (2)	−0.004 (2)	0.0003 (18)
C15	0.035 (2)	0.024 (2)	0.027 (2)	0.0056 (17)	−0.0011 (18)	−0.0046 (16)
C16	0.027 (2)	0.019 (2)	0.037 (2)	0.0056 (15)	0.0124 (18)	−0.0005 (16)
C17	0.030 (2)	0.020 (2)	0.039 (2)	0.0018 (16)	0.0105 (18)	−0.0001 (17)
C18	0.029 (2)	0.020 (2)	0.039 (2)	0.0005 (16)	0.0105 (18)	0.0004 (17)
C19	0.033 (2)	0.020 (2)	0.036 (2)	−0.0053 (16)	0.0123 (18)	−0.0043 (17)
C20	0.025 (2)	0.025 (2)	0.074 (3)	−0.0038 (17)	0.023 (2)	−0.005 (2)
C21	0.019 (2)	0.024 (2)	0.031 (2)	−0.0005 (15)	0.0100 (16)	0.0025 (16)
N4	0.0213 (17)	0.0140 (15)	0.0293 (17)	−0.0003 (12)	0.0092 (13)	−0.0001 (13)
C23	0.025 (2)	0.023 (2)	0.071 (3)	−0.0001 (17)	0.023 (2)	−0.011 (2)
N1	0.0244 (17)	0.0182 (16)	0.0264 (17)	0.0042 (12)	0.0104 (14)	0.0006 (13)
N2	0.0265 (17)	0.0127 (16)	0.0304 (17)	0.0036 (12)	0.0086 (14)	0.0024 (12)
N3	0.0238 (17)	0.0149 (16)	0.0341 (17)	−0.0021 (13)	0.0099 (14)	−0.0015 (13)
O1	0.0282 (14)	0.0136 (12)	0.0333 (14)	0.0018 (10)	0.0196 (11)	0.0008 (11)
O2	0.0385 (16)	0.0226 (14)	0.0284 (14)	0.0093 (11)	0.0246 (12)	0.0068 (11)
O1W	0.0230 (15)	0.0157 (14)	0.0277 (15)	0.0028 (11)	0.0138 (12)	−0.0024 (11)
O3	0.0388 (16)	0.0215 (13)	0.0276 (14)	0.0028 (11)	0.0226 (12)	0.0014 (11)
O4	0.0309 (15)	0.0202 (14)	0.0316 (14)	0.0091 (11)	0.0178 (12)	0.0062 (11)
Ni1	0.0259 (3)	0.0180 (2)	0.0269 (3)	0.0016 (2)	0.0137 (2)	0.0008 (2)

### Geometric parameters (Å, °)

**Table:**

C1—O4	1.240 (4)	C16—C17	1.505 (5)
C1—O2	1.270 (4)	C16—H16A	0.9700
C1—C2	1.508 (4)	C16—H16B	0.9700
C2—C3	1.356 (4)	C17—C18	1.525 (4)
C2—C8	1.434 (4)	C17—H17A	0.9700
C3—C4	1.400 (4)	C17—H17B	0.9700
C3—H3	0.9300	C18—C19	1.515 (4)
C4—C5	1.375 (4)	C18—H18A	0.9700
C4—H4	0.9300	C18—H18B	0.9700
C5—C7	1.420 (4)	C19—N3	1.466 (4)
C5—C6	1.500 (4)	C19—H19A	0.9700
C6—O3	1.240 (4)	C19—H19B	0.9700
C6—O1	1.279 (4)	C20—C23	1.338 (5)
C7—C12	1.421 (4)	C20—N3	1.368 (4)
C7—C8	1.424 (4)	C20—H20	0.9300
C8—C9	1.412 (4)	C21—N4	1.306 (4)
C9—C10	1.356 (4)	C21—N3	1.341 (4)
C9—H9	0.9300	C21—H21	0.9300
C10—C11	1.392 (5)	N4—C23	1.367 (4)
C10—H10	0.9300	N4—Ni1^i^	2.099 (3)
C11—C12	1.347 (4)	C23—H23	0.9300
C11—H11	0.9300	O1—Ni1^ii^	2.116 (2)
C12—H12	0.9300	O1W—HW12	0.819 (16)
C13—C14	1.343 (5)	O1W—HW11	0.83 (4)
C13—N1	1.355 (4)	O3—Ni1^ii^	2.347 (2)
C13—H13	0.9300	Ni1—O1W	2.125 (2)
C14—N2	1.363 (4)	Ni1—O2	2.040 (2)
C14—H14	0.9300	Ni1—O1^iii^	2.116 (2)
C15—N1	1.299 (4)	Ni1—O3^iii^	2.347 (2)
C15—N2	1.334 (4)	Ni1—N1	2.060 (3)
C15—H15	0.9300	Ni1—N4^iv^	2.099 (3)
C16—N2	1.472 (4)		
			
O4—C1—O2	124.8 (3)	H17A—C17—H17B	107.9
O4—C1—C2	120.4 (3)	C19—C18—C17	110.8 (3)
O2—C1—C2	114.8 (3)	C19—C18—H18A	109.5
C3—C2—C8	119.5 (3)	C17—C18—H18A	109.5
C3—C2—C1	118.9 (3)	C19—C18—H18B	109.5
C8—C2—C1	121.5 (3)	C17—C18—H18B	109.5
C2—C3—C4	121.6 (3)	H18A—C18—H18B	108.1
C2—C3—H3	119.2	N3—C19—C18	113.0 (3)
C4—C3—H3	119.2	N3—C19—H19A	109.0
C5—C4—C3	121.1 (3)	C18—C19—H19A	109.0
C5—C4—H4	119.5	N3—C19—H19B	109.0
C3—C4—H4	119.5	C18—C19—H19B	109.0
C4—C5—C7	119.2 (3)	H19A—C19—H19B	107.8
C4—C5—C6	119.1 (3)	C23—C20—N3	106.2 (3)
C7—C5—C6	121.7 (3)	C23—C20—H20	126.9
O3—C6—O1	120.7 (3)	N3—C20—H20	126.9
O3—C6—C5	121.2 (3)	N4—C21—N3	112.7 (3)
O1—C6—C5	118.1 (3)	N4—C21—H21	123.6
C5—C7—C12	122.5 (3)	N3—C21—H21	123.6
C5—C7—C8	119.6 (3)	C21—N4—C23	104.3 (3)
C12—C7—C8	117.8 (3)	C21—N4—Ni1^i^	128.0 (2)
C9—C8—C7	118.6 (3)	C23—N4—Ni1^i^	127.4 (2)
C9—C8—C2	122.4 (3)	C20—C23—N4	110.8 (3)
C7—C8—C2	118.9 (3)	C20—C23—H23	124.6
C10—C9—C8	121.5 (3)	N4—C23—H23	124.6
C10—C9—H9	119.2	C15—N1—C13	104.8 (3)
C8—C9—H9	119.2	C15—N1—Ni1	126.0 (2)
C9—C10—C11	119.7 (3)	C13—N1—Ni1	129.1 (2)
C9—C10—H10	120.2	C15—N2—C14	105.8 (3)
C11—C10—H10	120.2	C15—N2—C16	126.7 (3)
C12—C11—C10	121.3 (3)	C14—N2—C16	127.5 (3)
C12—C11—H11	119.4	C21—N3—C20	106.0 (3)
C10—C11—H11	119.4	C21—N3—C19	125.8 (3)
C11—C12—C7	121.1 (3)	C20—N3—C19	128.1 (3)
C11—C12—H12	119.5	C6—O1—Ni1^ii^	94.87 (19)
C7—C12—H12	119.5	C1—O2—Ni1	130.1 (2)
C14—C13—N1	110.2 (3)	Ni1—O1W—HW12	126 (2)
C14—C13—H13	124.9	Ni1—O1W—HW11	97 (3)
N1—C13—H13	124.9	HW12—O1W—HW11	110 (2)
C13—C14—N2	106.3 (3)	C6—O3—Ni1^ii^	85.3 (2)
C13—C14—H14	126.9	O2—Ni1—N1	85.93 (10)
N2—C14—H14	126.9	O2—Ni1—N4^iv^	102.65 (10)
N1—C15—N2	112.9 (3)	N1—Ni1—N4^iv^	96.70 (11)
N1—C15—H15	123.6	O2—Ni1—O1^iii^	159.34 (9)
N2—C15—H15	123.6	N1—Ni1—O1^iii^	94.01 (10)
N2—C16—C17	112.4 (3)	N4^iv^—Ni1—O1^iii^	97.87 (10)
N2—C16—H16A	109.1	O2—Ni1—O1W	85.20 (9)
C17—C16—H16A	109.1	N1—Ni1—O1W	169.33 (10)
N2—C16—H16B	109.1	N4^iv^—Ni1—O1W	91.03 (10)
C17—C16—H16B	109.1	O1^iii^—Ni1—O1W	92.20 (9)
H16A—C16—H16B	107.9	O2—Ni1—O3^iii^	100.97 (9)
C16—C17—C18	111.9 (3)	N1—Ni1—O3^iii^	86.35 (10)
C16—C17—H17A	109.2	N4^iv^—Ni1—O3^iii^	156.33 (9)
C18—C17—H17A	109.2	O1^iii^—Ni1—O3^iii^	58.47 (8)
C16—C17—H17B	109.2	O1W—Ni1—O3^iii^	89.53 (8)
C18—C17—H17B	109.2		

Symmetry codes: (i) *x*, *y*+1, *z*; (ii) *x*−1/2, −*y*+1/2, *z*+1/2; (iii) *x*+1/2, −*y*+1/2, *z*−1/2; (iv) *x*, *y*−1, *z*.

### Hydrogen-bond geometry (Å, °)

**Table:**

*D*—H···*A*	*D*—H	H···*A*	*D*···*A*	*D*—H···*A*
O1W—HW12···O1^v^	0.819 (16)	1.847 (18)	2.661 (3)	172 (3)
O1W—HW11···O4	0.83 (4)	1.83 (4)	2.651 (3)	169 (3)

Symmetry codes: (v) −*x*+1/2, *y*−1/2, −*z*+1/2.
